# Ross procedure after prior aortic valve intervention: Outcomes from the North American Ross Consortium database

**DOI:** 10.1016/j.xjon.2025.101565

**Published:** 2025-12-22

**Authors:** Cody W. Dorton, Taylor Pickering, Kyle A. McCullough, John B. Eisenga, Tanushree Prasad, Mary C. Parro, Emma B. Galvanek, Amro Alsaid, Zuyue Wang, Justin M. Schaffer, J. Michael DiMaio, Nimesh Desai, Scott C. DeRoo, Christopher R. Burke, Christopher K. Mehta, S. Christopher Malaisrie, William T. Brinkman, Katherine B. Harrington, North American Ross Consortium Working Group

**Affiliations:** a Department of Cardiovascular Research, Baylor Scott & White Research Institute, Plano, Tex; b Department of Biostatistics, Baylor Scott & White Research Institute, Plano, Tex; c Department of Cardiac Imaging, Baylor Scott & White – The Heart Hospital Plano, Plano, Tex; d Department of Cardiac Surgery, Baylor Scott & White – The Heart Hospital Plano, Plano, Tex; e Department of Biomedical Engineering, Texas A&M University, College Station, Tex; f Division of Cardiovascular Surgery, University of Pennsylvania, Philadelphia, Pa; g Division of Cardiothoracic Surgery, University of Washington, Seattle, Wash; h Division of Cardiac Surgery, Northwestern University, Chicago, Ill

**Keywords:** North American Ross Consortium, Ross procedure, pulmonary autograft procedure, multicenter, retrospective outcomes

## Abstract

**Background:**

Excellent results for the Ross procedure have been demonstrated in selected, single-center series. Outcomes for higher-risk patients, including those with prior aortic valve intervention, are not well described, however.

**Methods:**

This multicenter, retrospective analysis of adult recipients of the Ross procedure between 1994 and 2025 at 4 high-volume aortic centers compared patients with prior surgical aortic valve intervention (SAVI) and those with no prior aortic valve intervention. The primary outcome was operative mortality. Secondary outcomes included perioperative outcomes, longitudinal mortality, and reintervention.

**Results:**

The study cohort comprised 594 patients, including 29 with prior SAVI and 565 without prior SAVI. The median age at surgery was 38 years (interquartile range [IQR], 32-48 years) for the SAVI group and 42 years (IQR, 32-51 years) for the non-SAVI group (*P* = .16). Racial distribution, body mass index, and male sex were comparable between the 2 groups. Rates of endocarditis were higher in the SAVI group compared to the non-SAVI group (treated: 6.9% vs 4.3%; active: 10.3% vs 1.4%; *P* = .012). There was 1 (3.5%) operative death in the SAVI group and 4 (0.7%) operative deaths the non-SAVI group (*P* = .22). Five-year Kaplan-Meier survival was higher in the non-SAVI group (log-rank *P* = .004), although no causes of death were cardiac in nature.

**Conclusions:**

Cardiac causes of death were absent in those with prior SAVI following the Ross procedure, suggesting that the Ross procedure can be safe in these patients when performed by skilled, experienced surgeons. Further studies are needed to more fully assess Ross procedure outcomes in patients with prior SAVI.

**Figure:**
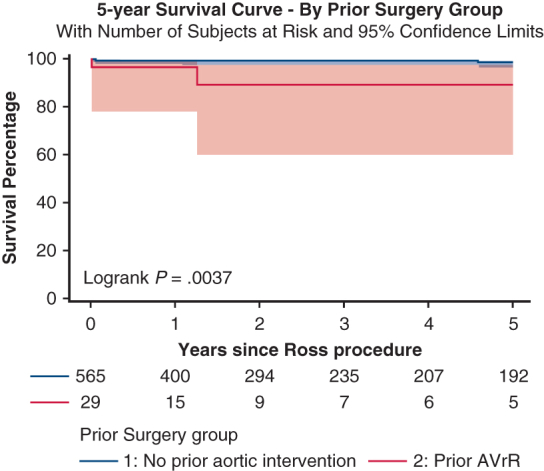
Five-year Kaplan–Meier survival estimates stratified by history of prior aortic valve intervention. Shaded area represents 95% confidence interval.

**Central Message**

The Ross procedure may be performed successfully in patients with prior aortic valve interventions.

**Perspective**

Previous aortic valve intervention may complicate the Ross procedure in adults. In this series, we describe outcomes following the Ross procedure in those with previous aortic valve repair/replacement.

First described by Dr Donald Ross in 1967, replacement of the aortic valve using the autologous pulmonic valve (the Ross procedure) provides a surgical solution for aortic valve disease without the challenges associated with mechanical or biologic valve prostheses.^1^^,^^2^ The theoretical advantages of the Ross procedure—implantation of a living valve substitute with native hemodynamics, resistance to infection, and avoidance of lifelong anticoagulation—have long appealed to both surgeons and patients, particularly younger adults with a long life expectancy. Initial enthusiasm for the Ross procedure reached its height in the 1990s and subsequently waned^3^; however, recent investigations from experienced centers have demonstrated excellent operative outcomes, long-term durability, and survival approximating that of the general population, fueling a renaissance of this technique in adult populations.^4^, ^5^, ^6^, ^7^, ^8^, ^9^

Despite this accumulating evidence, the majority of Ross series exclude patients with prior surgical aortic valve intervention (SAVI).^4^^,^^8^^,^^10^ Patients with prior SAVI compose an increasingly prevalent subgroup owing to the growth of congenital heart disease survivor populations, yet the impact of prior SAVI on Ross procedure outcomes remains poorly defined. It was hypothesized that prior SAVI would be associated with higher perioperative risk but similar long-term survival compared to patients without prior SAVI.

## Methods

### The North American Ross Consortium

The North American Ross Consortium database represents a multicenter collaboration across 4 high-volume thoracic aortic centers: Baylor Scott & White–The Heart Hospital, University of Washington, Northwestern University, and University of Pennsylvania. Patients age ≥18 years who underwent the Ross procedure were eligible for enrollment. Historical Ross recipients from July 1, 1994, through April 30, 2024, were enrolled retrospectively. Individual patient chart reviews were conducted, and relevant available data was entered into the database. Prospective collection of highly granular data began October 1, 2023, to identify specific outcomes and best practices in the care of Ross patients.

Patients were stratified based on prior aortic valve intervention. The SAVI group included patients who had previously undergone aortic valve repair (AVr), aortic valve replacement (AVR), or valve-sparing root replacement. Patients with no prior aortic valve intervention, as well as those with prior transcatheter intervention such as balloon aortic valvuloplasty (BAV), were classified as having no prior intervention (non-SAVI group). The primary outcome was operative mortality, defined as mortality within 30 days of surgery. Secondary outcomes included perioperative outcomes, longitudinal mortality, and reintervention. All techniques for the Ross procedure were eligible for inclusion. Techniques for autograft harvest, anastomosis, myocardial protection, and use of annular and sinotubular junction support were at the discretion of the primary surgeon.

### Data Management and Analysis

Study data were collected and managed using REDCap electronic data capture tools hosted at Baylor Scott & White–The Heart Hospital. REDCap is a secure, web-based software platform designed to support data capture for research studies, providing (1) an intuitive interface for validated data capture; (2) audit trails for tracking data manipulation and export procedures; (3) automated export procedures for seamless data downloads to common statistical packages; and (4) procedures for data integration and interoperability with external sources.^11^^,^^12^

Data were tabulated using descriptive statistics. Categorical variables are presented as count with percentage. Continuous variables were assessed for normality and are presented as mean ± SD for normally distributed data or as median (interquartile range [IQR]) as appropriate. Continuous variables were compared using the Wilcoxon rank-sum test, and categorical variables were compared using the χ^2^ test or Fisher exact test, as appropriate. Analyses were performed using R version 4.4.0 (R Foundation for Statistical Computing).

This study was reviewed and approved by the Baylor Scott & White Research Institute's Institutional Review Board (IRB# 023-286; approved July 30, 2023). Site-specific Institutional Review Board approval and a waiver of written informed consent were granted for retrospective patient data collection. All patients enrolled prospectively provided informed consent prior to undergoing a planned Ross procedure. Informed consent was waived for retrospectively enrolled patients.

## Results

### Patient Characteristics

The study cohort comprised 594 patients, including 29 with prior SAVI and 565 with no prior SAVI. The median age at surgery was 38 years (IQR, 32-46 years) for the SAVI group and 42 years (IQR, 32-51 years) for the non-SAVI group (*P* = .16). Table 1 summarizes demographic and clinical characteristics for the SAVI and non-SAVI groups. Sex distribution, racial distribution, and body mass index were comparable in the 2 groups. Rates of endocarditis, both previously treated and active, were significantly higher in the SAVI group compared to the non-SAVI group (treated: 6.9% [n = 2] vs 4.3% [n = 24]; active: 10.3% [n = 3] vs 1.4% [n = 8]; overall *P* = .012). Hypertension was more prevalent in the non-SAVI group (36.3% [n = 205] vs 20.7% [n = 6]), although the difference was not statistically significant (*P* = .087).

**Table 1:** Demographic and clinical characteristics

Characteristic	SAVI group (N = 29)	Non-SAVI group (N = 565)	Total cohort (N = 594)	*P* value
Age at surgery, y, median [IQR]	38 [32-46]	42 [33-51]	42 [33-51]	.16
Male sex, n (%)	19 (65.5)	357 (70.0)	393 (69.7)	.63
BMI, kg/m^2^, median [IQR]	27.6 [24.9-30.7]	27.5 [24.2-31.7]	27.5 [24.2-31.5]	.76
Race, n (%)				.62
American Indian/Alaskan-Native	0 (0.0)	2 (0.4)	2 (0.3)	
Asian	2 (6.9)	20 (3.5)	22 (3.7)	
Black/African American	1 (3.4)	13 (2.3)	14 (2.4)	
Native-Hawaiian/Other Pacific Islander	0 (0.0)	3 (0.5)	3 (0.5)	
White	24 (82.8)	479 (84.8)	503 (84.7)	
Other	2 (6.9)	48 (8.5)	50 (8.4)	
Dialysis, n (%)	0 (0.0)	2 (0.4)	2 (0.3)	1.00
Hypertension, n (%)	6 (20.7)	205 (36.3)	211 (35.5)	.087
Atrial fibrillation, n (%)	1 (3.4)	19 (3.4)	20 (3.4)	1.00
Pacemaker, n (%)	0 (0.0)	9 (1.6)	9 (1.5)	1.00
Endocarditis, n (%)				**.012**
Treated	2 (6.9)	24 (4.3)	26 (4.4)	
Active	3 (10.3)	8 (1.4)	11 (1.9)	
Prior stroke, n (%)	1 (3.4)	6 (1.1)	7 (1.2)	.30
Prior MI, n (%)	0 (0.0)	11 (1.9)	11 (1.9)	1.00
CAD, n (%)	1 (3.4)	34 (6.0)	35 (5.9)	1.00
CABG, n (%)	0 (0.0)	3 (0.5)	3 (0.5)	1.00
PCI, n (%)	0 (0.0)	7 (1.2)	7 (1.2)	1.00
NYHA class, n (%)				.94
I	3 (10.3)	76 (13.5)	79 (13.3)	
II	8 (27.6)	161 (28.5)	169 (28.5)	
III	5 (17.2)	108 (19.1)	113 (19.0)	
IV	0 (0.0)	18 (3.2)	18 (3.2)	
Tobacco use, n (%)				.053
Never	22 (75.9)	355 (62.8)	377 (62.8)	
Current	5 (17.2)	76 (13.5)	81 (13.6)	
Former	1 (3.4)	121 (21.4)	122 (20.5)	
Unknown	1 (3.4)	13 (2.3)	14 (2.4)	
Valve lesion, n (%)				
Isolated AS	17 (58.6)	353 (62.5)	370 (62.3)	.68
Isolated AI	1 (3.4)	39 (6.9)	40 (6.7)	.71
Mixed AS/AI	8 (27.6)	180 (31.9)	188 (31.6)	.63
Urgency of surgery, n (%)				.81
Elective	26 (89.7)	509 (90.1)	535 (90.1)	
Urgent	1 (3.4)	24 (4.2)	25 (4.2)	
Emergent	1 (3.4)	12 (2.1)	13 (2.2)	
Missing	1 (3.4)	20 (3.5)	21 (3.5)	
Preoperative LVEF, n (%)				.20
≤35	0 (0.0)	5 (1.0)	5 (1.0)	
36-54	0 (0.0)	56 (11.5)	56 (10.9)	
≥55	26 (100)	426 (87.5)	452 (88.1)	
Missing	3 (10.3)	78 (13.8)	81 (13.6)	

Bold type indicates statistical significance.

*SAVI*, Surgical aortic valve intervention; *IQR*, interquartile range; *BMI*, body mass index; *MI*, myocardial infarction; *CAD*, coronary artery disease; *CABG*, coronary artery bypass grafting; *PCI*, percutaneous coronary intervention; *NYHA*, New York Heart Association functional classification; *AS*, aortic stenosis; *AI*, aortic insufficiency; *LVEF*, left ventricular ejection fraction.

### Operative Data

Intraoperative and perioperative characteristics are presented in Table 2. Median cardiopulmonary bypass time and aortic cross-clamp times were longer in the SAVI group compared to the non-SAVI group (bypass: 248 [IQR, 211-277] minutes vs 212 [IQR, 187-246] minutes [*P* < .001]; cross-clamp: 203 [IQR, 181-242] minutes vs 186 [IQR, 167-213] minutes [*P* = .001]). The intraoperative aortic annulus size was smaller in the SAVI group (median, 23.5 [IQR, 23-28] mm vs 27 [IQR, 24-29] mm; *P* = .011). Other operative characteristics, including concomitant procedure rates, use of hemiarch replacement, and use of circulatory arrest, were comparable in the 2 groups.

**Table 2:** Intraoperative and perioperative characteristics

Characteristic	SAVI group (N = 29)	Non-SAVI group (N = 565)	Total cohort (N= 594)	*P* value
CPB time, min, median [IQR]	248 [211-277]	212 [187-246]	213 [189-249]	**<.001**
Cross-clamp time, min, median [IQR]	203 [181-242]	186 [167-213]	188 [167-214]	**.001**
Hemiarch replacement, n (%)	2 (6.9)	56 (9.9)	58 (9.8)	1.00
Circulatory arrest, n (%)	4 (13.8)	62 (11.0)	66 (11.1)	.55
Ascending aortic replacement, n (%)	13 (44.8)	251 (44.4)	264 (44.4)	.97
Aortic annulus size, mm, median [IQR]	23.5 [23-28]	27 [24-29]	27 [24-29]	**.011**
Concomitant-procedures, n (%)				1.00
CABG	0 (0.0)	17 (3.0)	17 (2.9)	
Mitral valve intervention	0 (0.0)	7 (1.2)	7 (1.2)	
Tricuspid valve intervention	0 (0.0)	3 (0.5)	3 (0.5)	
ICU length of stay, d, median [IQR]	3.4 [1.7-4.1]	1.9 [1.0-3.6]	1.9 [1.0-3.8]	.086
Hospital length of stay, d, median [IQR]	6 [4.5-7]	5 [4-7]	6 [4-7]	.74

Bold type indicates statistical significance.

*SAVI*, Surgical aortic valve intervention; *CPB*, cardiopulmonary bypass; *IQR*, interquartile range; *CABG*, coronary artery bypass grafting; *ICU*, intensive care unit.

### Operative Outcomes

Operative outcomes are summarized in Table 3. There was 1 (3.5%) operative death in the SAVI group and 4 (0.7%) operative deaths in the non-SAVI group (*P* = .22). In-hospital reoperation rates were similar in the 2 groups, with 2 (6.9%) in the SAVI group and 18 (3.2%) in the non-SAVI group (*P* = .26). Fresh frozen plasma use was higher in the SAVI group (20.7% [n = 6] vs 6.3% [n = 35]; *P* = .011). Rates of postoperative myocardial infarction, pacemaker implantation, deep sternal wound infection, and delirium were not significantly different between the groups. And all other operative outcomes were similar in the 2 groups.

**Table 3:** Operative outcomes

Characteristic	SAVI group (N = 29), n (%)	Non-SAVI group (N = 565), n (%)	Total cohort (N = 594), n (%)	*P* value
In-hospital reoperation	2 (6.9)	18 (3.2)	20 (3.4)	.26
Reason for reoperation				
Bleeding	0 (0.0)	13 (4.0)	13 (3.8)	1.00
Homograft dysfunction	0 (0.0)	1 (0.3)	1 (0.3)	1.00
Autograft dysfunction	0 (0.0)	1 (0.3)	1 (0.3)	1.00
Heart failure	2 (13.3)	2 (0.6)	4 (1.2)	**.010**
Acute kidney injury	2 (6.9)	37 (6.6)	39 (6.6)	1.00
Renal failure requiring dialysis	0 (0.0)	10 (1.8)	10 (1.7)	1.00
Prolonged ventilation	1 (4.8)	18 (6.2)	19 (6.1)	1.00
Tracheostomy	0 (0.0)	4 (0.7)	4 (0.7)	1.00
Reintubation	0 (0.0)	15 (2.7)	15 (2.6)	1.00
Pneumonia	0 (0.0)	16 (2.9)	16 (2.7)	1.00
Transient ischemic attack	0 (0.0)	1 (0.2)	1 (0.2)	1.00
Stroke	1 (3.4)	5 (0.9)	6 (1.0)	.26
Delirium	3 (10.3)	15 (2.7)	18 (3.1)	.053
Atrial fibrillation/flutter	3 (10.3)	84 (15.0)	87 (14.8)	.79
AICD/PPM	3 (10.3)	17 (3.0)	20 (3.4)	.070
Myocardial infarction	1 (3.4)	2 (0.4)	3 (0.5)	.14
Deep sternal wound infection	1 (3.4)	1 (0.2)	2 (0.3)	.10
Operative mortality	1 (3.5)	4 (0.7)	5 (0.8)	.22

Bold type indicates statistical significance.

*SAVI,* Surgical aortic valve intervention; *AICD*, automatic implantable cardioverter-defibrillator; *PPM*, permanent pacemaker.

### Long-Term Outcomes

**Figure 1:**
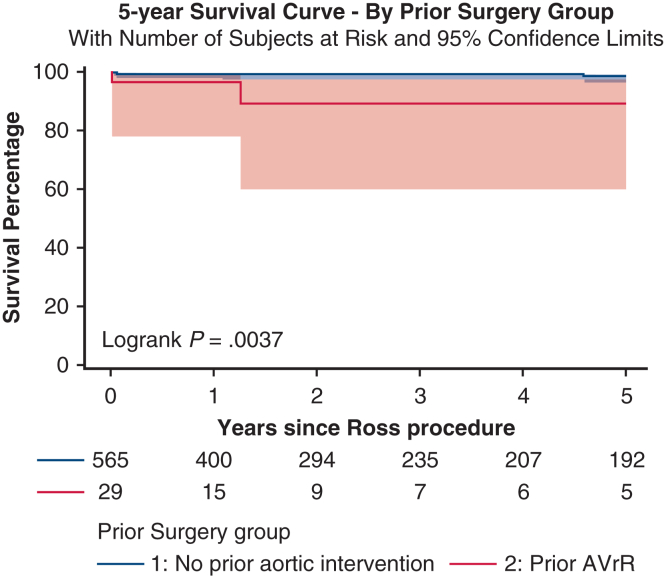
Five-year Kaplan-Meier survival estimates stratified by history of prior surgical aortic valve intervention. Shaded areas represent 95% confidence interval.

Postdischarge clinical follow-up was available for 99.5% of patients, and the median duration of follow-up was 12.2 months (IQR, 6.5-34.0 months) for the SAVI group and 25.4 months (IQR, 9.7-160.4 months) for the non-SAVI group (*P* = .046). Rates of readmission, reintervention, major adverse cardiovascular events (combination of myocardial infarction, stroke, and readmission for heart failure or valve dysfunction), and postdischarge complications were similar in the 2 group (Table 4). Five-year Kaplan-Meier survival was higher in the non-SAVI group (*P* = .004, log-rank test; Figure 1), and all-cause mortality was higher in the SAVI group (13.8% [n = 4] vs 5.7% [n = 32]; *P* = .008). Causes of death in the SAVI group included cancer, motor vehicle collision, heparin-induced thrombocytopenia and thrombosis following cardiopulmonary bypass, and unknown (all n = 1).

**Table 4:** Long-term outcomes

Outcome	SAVI group (N = 29)	Non-SAVI group (N = 565)	Total (N = 594)	*P* value
Length of follow-up, mo, median [IQR]	12.2 [6.5-34.0]	25.4 [9.7-160.4]	24.7 [9.2-158.3]	**.046**
Reason for readmission, n (%)				
Any-cause	9 (31.0)	204 (36.1)	213 (35.9)	.58
Aortic complications	0 (0.0)	14 (2.5)	14 (2.5)	1.00
Arrythmia/heart block	2 (6.9)	61 (10.8)	63 (10.6)	.76
Congestive heart failure	0 (0.0)	12 (2.1)	12 (2.0)	1.00
Pericardial effusion/tamponade	0 (0.0)	6 (1.1)	6 (1.0)	1.00
Pleural effusion requiring intervention	0 (0.0)	2 (0.4)	2 (0.3)	1.00
Valve dysfunction	3 (10.3)	66 (11.7)	69 (11.6)	1.00
Procedure performed during readmission, n (%)	6 (20.7)	153 (27.1)	159 (26.8)	.45
Valve-related reintervention, n (%)	4 (3.4)	67 (11.9)	71 (12.0)	.75
Autograft reimplantation	0 (0.0)	7 (1.2)	7 (1.3)	1.00
Autograft remodeling	0 (0.0)	1 (0.2)	1 (0.2)	1.00
AVR	1 (3.4)	36 (6.4)	37 (6.2)	1.00
Homograft replacement	0 (0.0)	23 (4.1)	23 (3.9)	1.00
Stroke after discharge, n (%)	0 (0.0)	15 (2.7)	15 (2.5)	1.00
Pseudoaneurysm after discharge, n (%)	0 (0.0)	13 (2.3)	13 (2.2)	1.00
Myocardial infarction after discharge, n (%)	0 (0.0)	5 (0.9)	5 (0.8)	1.00
Bleeding after discharge, n (%)	0 (0.0)	19 (3.4)	19 (3.2)	.62
Endocarditis, n (%)	0 (0.0)	23 (4.1)	23 (3.9)	.62
Arrhythmia after discharge, n (%)	2 (6.9)	98 (17.3)	100 (16.8)	.20
Atrial fibrillation	0 (0.0)	37 (6.5)	37 (6.2)	.25
Atrial flutter	0 (0.0)	15 (2.7)	15 (2.7)	1.00
Bradycardia	1 (3.4)	35 (6.2)	36 (6.1)	1.00
AV block	1 (3.4)	28 (5.0)	29 (4.9)	1.00
Arrhythmia intervention: cardioversion, n (%)	0 (0.0)	17 (3.0)	17 (2.9)	1.00
PPM placed, n (%)	1 (3.4)	22 (3.9)	23 (3.9)	1.00
MACE, n (%)	6 (20.7)	129 (22.8)	135 (22.7)	.79

Bold type indicates statistical significance.

*SAVI*, Surgical aortic valve intervention; *IQR*, interquartile range; *AVR*, aortic valve replacement; *AV*, atrioventricular; *PPM*, permanent pacemaker; *MACE*, major adverse cardiovascular event.

## Discussion

Multiple analyses have demonstrated that the Ross procedure provides survival and durability advantages over mechanical or bioprosthetic AVR. A systematic review by Mazine and colleagues reported that the Ross procedure was associated with a 48% relative reduction in all-cause mortality and lower risks of stroke and major bleeding compared to mechanical AVR.^4^ Buratto and colleagues^13^ similarly found higher 20-year survival following the Ross procedure compared to mechanical AVR, with the survival of Ross patients closely approximating that of an age- and sex-matched general population. McClure and colleagues^7^ confirmed these findings, demonstrating lower mortality and improved freedom from thromboembolic and bleeding complications for Ross recipients. Finally, Etnel and colleagues, in the most comprehensive analysis reported to date, found that the 30-year life expectancy following the Ross procedure approached 97% of that in the age- and sex-matched general population, with lower rates of thromboembolic events, bleeding, and endocarditis compared to conventional prostheses.^10^ These findings support the Ross procedure as the preferred AVR strategy for young and middle-aged adults when performed by experienced surgeons.

Prior SAVI has been a variable of interest in the Ross literature, but its impact on outcomes has been poorly defined. In their meta-regression, Etnel and colleagues^10^ identified prior SAVI as a possible predictor of increased reintervention risk, and Notenboom and colleagues^14^ included prior SAVI patients in their series; however, outcomes were not stratified for this subgroup. To our knowledge, our study is the first multicenter series to describe survival outcomes in this unique population.

In our analysis, prior SAVI did not significantly impact operative mortality but did impact long-term survival. Patients with prior SAVI demonstrated reduced survival compared to those without prior SAVI, with survival differences evident both at and beyond 5 years. However, the lack of cardiac causes of death suggests that this mortality rate likely is unrelated to the Ross procedure but rather is impacted by external factors. Operative mortality was nonsignificantly higher among AVr and AVR patients compared to those with prior BAV or no prior intervention. Further, rates of valve-related reintervention, annular dilation, and subsequent aortic insufficiency were similar in the SAVI and non-SAVI groups, suggesting that prior intervention did not adversely affect autograft durability in the short-to mid-term. Additionally, despite increased operative complexity and associated longer bypass and aortic cross-clamp times, rates of postoperative complications, including reoperation, transfusion requirements, acute kidney injury, and stroke, were similar in the 2 groups.

Despite the increased technical complexity and increased intensive care unit length of stay in the prior SAVI group, perioperative outcomes were similar in the 2 groups. Mortality, while numerically higher in the SAVI group, was related primarily to noncardiac etiologies. These findings suggest that when performed by experienced surgeons in high-volume centers, the Ross procedure can be executed safely even in the reoperative setting. Early autograft durability appeared to be preserved, with no significant between-group differences in valve-related reintervention, annular dilation, or autograft function. This is consistent with prior studies demonstrating excellent early and mid-term autograft performance.^4^^,^^13^^,^^15^ Nonetheless, it should be noted that progressive annular and root dilation remains the predominant mechanism of late Ross failure, with most autograft reintervention occurring beyond the first decade of follow-up.^10^^,^^14^^,^^16^ As such, ongoing long-term surveillance will be critical to fully assess autograft durability in this complex subgroup.

Our findings carry important implications. In patients with prior BAV, the Ross procedure can be performed safely, with similar short- and long-term results in patients with an intervention-naïve aortic valve. In patients with prior SAVI, the Ross procedure is viable option with good outcomes when performed by an experienced surgeon in a high-volume center.

### Limitations

This study has several limitations. The number of patients in the prior SAVI group was relatively small, limiting the statistical power for some comparisons. As a retrospective analysis, this study is subject to the inherent limitations and biases of such studies. Unequal representation of participating centers in terms of patient contributions also should be noted, which may introduce center-level variability in outcomes. Additionally, the study period spanned 3 decades, during which important technical procedural modifications were adopted; therefore, outcomes of the earliest patients might not fully reflect those achievable with contemporary surgical practice.

## Conclusions

The Ross procedure can be performed safely in selected patients with prior SAVI. While the SAVI patients exhibited increased operative complexity and late noncardiac mortality, acceptable outcomes can be achieved when these procedures are performed by highly experienced aortic surgeons. Proceeding with the Ross procedure in this population can be considered by experienced Ross surgeons at centers with advanced aortic expertise. Further studies are needed to refine patient selection and optimize surgical strategies for this challenging subgroup.

### Audio

You can listen to the discussion audio of this article by going to the Supplemental Materials.

## Conflict of Interest Statement

The authors reported no conflicts of interest.

The *Journal* policy requires editors and reviewers to disclose conflicts of interest and to decline handling or reviewing manuscripts for which they may have a conflict of interest. The editors and reviewers of this article have no conflicts of interest.

## Supplementary Data

Audio 1 This is an audio recording of the presentation of the associated abstract and discussion. The abstract presentation ends at 5:00 and the discussion begins at 5:05.
